# Dual functionality of *Trichoderma*: Biocontrol of *Sclerotinia sclerotiorum* and biostimulant of cotton plants

**DOI:** 10.3389/fpls.2022.983127

**Published:** 2022-10-07

**Authors:** Lucas Guedes Silva, Renato Cintra Camargo, Gabriel Moura Mascarin, Peterson Sylvio de Oliveira Nunes, Christopher Dunlap, Wagner Bettiol

**Affiliations:** ^1^ Department of Plant Protection, School of Agriculture, São Paulo State University (UNESP), Botucatu, Brazil; ^2^ Embrapa Environment, Laboratory of Environmental Microbiology, Jaguariúna, Brazil; ^3^ Department of Phytopathology, Federal University of Lavras, Lavras, Brazil; ^4^ Crop Bioprotection Research Unit, National Center for Agricultural Utilization Research, United States Department of Agriculture, Peoria, IL, United States

**Keywords:** bioprotectant, biofungicide, white mold, biofertilizer, phosphate solubilization ability

## Abstract

Microbial crop protection products based on *Trichoderma* have the ability to display multifunctional roles in plant protection, such as pathogen parasitism, enhance nutrient availability and stimulate plant growth, and these traits can be used to enhance the overall agronomic performance of a variety of crops. In the current study, we explored the multifunctional potential of two indigenous Brazilian strains of *Trichoderma* (*T. asperelloides* CMAA 1584 and *T. lentiforme* CMAA 1585) for their capability of controlling *Sclerotinia sclerotiorum*, a key plant pathogen of cotton, and for their ability of growth promotion in cotton plants (*Gossypium hirsutum*). Both strains were able to solubilize mineral phosphorus (CaHPO_4_), to release volatile organic compounds that impaired the mycelial growth of *S. sclerotiorum*, and to promote the growth of cotton plants under greenhouse conditions. In dual culture, *Trichoderma* strains reduced the growth rate and the number of sclerotia formed by *S. sclerotiorum*. By treating sclerotia with conidial suspensions of these *Trichoderma* strains, a strong inhibition of the myceliogenic germination was observed, as a result of the marked mycoparasitic activity exerted on the sclerotia. The parasitism over *S. sclerotiorum* was more effective with *T. asperelloides* CMAA 1584, whilst the biostimulant effects on cotton growth were more pronounced with *T. lentiforme* CMAA 1585, which also showed a higher capacity of phosphate solubilization. Thus*, T. asperelloides* CMAA 1584 displays higher efficiency in controlling *S. sclerotiorum*, while *T. lentiforme* CMAA 1585 is more suitable as a biostimulant due to its ability to promote growth in cotton plants. Overall, these *Trichoderma* strains may be used in mixture to provide both pathogen control and promotion of plant growth, and this strategy will support growers in minimizing the use of synthetic fertilizers and fungicides against white mold in cotton crops.

## Graphical Abstract

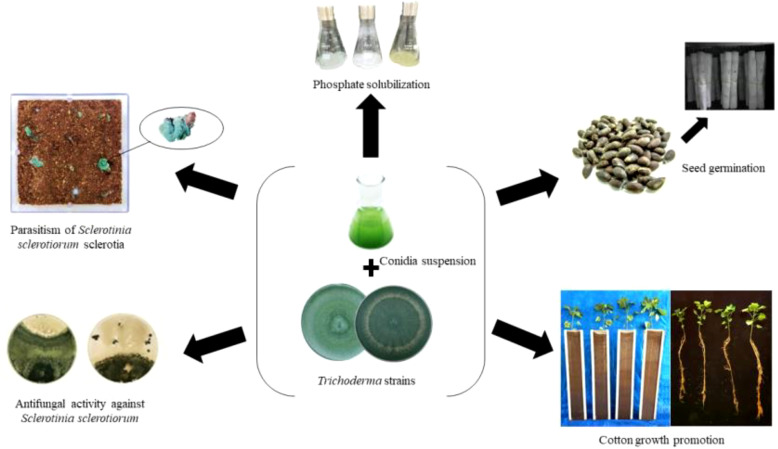

## Introduction

Cotton (*Gossypium hirsutum* L.) is the most important source of natural fibers in the world (Sivakumar et al., 2021; Wu et al., 2022). According to Tarazi et al. (2019), approximately 150 countries are directly involved in the cotton industrial chain, being an income source for more than 100 million families worldwide. Brazil stands out as the fourth largest producer of cotton worldwide, attaining a cultivated area of 1.6 million hectares with an estimated crop production of 6.7 million tons in 2021/22 (CONAB - Companhia Nacional de Abastecimento, 2022).

Since 1990s, the cotton growing area has dramatically expanded throughout the savannah Central-West region of Brazil (known as biome ‘Cerrado’), mainly due to breeding efforts for developing locally adapted high-yielding cultivars and improving agronomic practices (Morello et al., 2015; Silva Neto et al., 2016; Barroso et al., 2017; Silva et al., 2019). As a result, the cultivated area in the Mato Grosso State has increased by approximately 1,500% in the last 30 years (ABRAPA - Associação Brasileira dos Produtores de Algodão, 2021). However, the high incidence of pests and diseases remain inflicting high productivity losses, accounting for approximately 35% of production costs (IMEA - Instituto Mato-Grossense de Economia Agropecuária, 2019).

Among several diseases that limit cotton growth and yield, white mold, also known as Sclerotinia stem rot, caused by the ascomycete fungus *Sclerotinia sclerotiorum* (Lib.) de Bary (Ascomycota: Sclerotiniaceae), is one of the most devastating and yield-limiting diseases. This plant pathogen cause billions of dollars of crop losses and is of great economic importance to several agricultural and vegetable crops worldwide, notably including cotton and soybean (Boland and Hall, 1994; O’Sullivan et al., 2021). According to the Brazilian Ministry of Agriculture, Livestock and Food Supply (MAPA), *S. sclerotiorum* is considered one of the eight diseases/pests with the highest phytosanitary risk for Brazil (Brazil, 2015). This disease poses a serious threat to cotton plants at all phenological stages, and pathogen forms a dark-pigmented and hardened mycelial threads known as sclerotium capable of surviving for several years in soil (Tourneau, 1979; Schwartz and Singh, 2013). Symptoms associated with the white mold in cotton include wilt, necrosis and rotting of stems, bolls, petioles and leaves (Charchar et al., 1999; Suassuna et al., 2019).

Currently, the Mato Grosso State is responsible for approximately 71% of the Brazilian cotton production, following an integrated cropping system with soybean and corn (Silva et al., 2019; IMEA - Instituto Mato-Grossense de Economia Agropecuária, 2021). According to Meyer et al. (2020), *S. sclerotiorum* is endemic in approximately 27% of soybean production areas in Brazil, and 87% of cotton growing areas in Mato Grosso, in which cotton is cultivated as a second crop after soybean crop (IMEA - Instituto Mato-Grossense de Economia Agropecuária, 2021). Thus, the succession of susceptible crops to *S. sclerotiorum* has been responsible for the continuous increase of the incidence of white mold, leading to an overuse of chemical fungicides as a means to alleviate crop yield losses. However, over-reliance of broad-spectrum chemical fungicides poses a serious risk to the environment (Komárek et al., 2010), health of growers (Kniss, 2017), and accelerate the selection of resistant strains of *S. sclerotiorum* (Zhou et al., 2014), all of which require urgent alternative measures that include the development bio-rational solutions for the integrated management of white mold disease.

Despite the fact that chemical fungicides are effective in protecting plants from the white mold, their stand-alone use has inconsistent and unsatisfactory results. This is mainly due to difficulties in achieving adequate application coverage of the target pathogen, coupled with the best timing of application when ascospores are discharged (Meyer et al., 2014). Additionally, the lack of genetically resistant plants and the adoption of intensive management strategies based solely on synthetic fungicides have led to the development of resistant *S*. *sclerotiorum* strains to many chemical active ingredients (Liang et al., 2015; Mao et al., 2018). In this sense, it is imperative to explore alternative measures such as biological control strategies against this cosmopolitan plant pathogen (Bettiol et al., 2021).

Among biological control agents, *Trichoderma* spp. are considered effective in controlling *S. sclerotiorum* across several crops (Li et al., 2005; Sharma and Sain, 2010; Elias et al., 2016; Sumida et al., 2018), including cotton. The efficacy of using the necrotrophic mycoparasite *Trichoderma* in the management of *S. sclerotiorum* is related to its ability to parasitize and degrade sclerotia, resulting in an inversely proportional relationship between the frequency of *Trichoderma* application and the viability of sclerotia in the soil (Ferraz et al., 2011; Geraldine et al., 2013; Smolińska and Kowalska, 2018). Notably, *Trichoderma* spp. antagonize a myriad of plant pathogens by distinct mechanisms of action (Lorito et al., 2010; Druzhinina et al., 2011; Hermosa et al., 2012; Monte et al., 2019; Monte and Hermosa, 2021). Furthermore, due to the plasticity of their genomes in expressing multiple ecological functions and diverse biochemical machinery, several *Trichoderma* species promote plant growth (Rubio et al., 2017; Monte et al., 2019; Monte and Hermosa, 2021) and induce plant defenses against biotic and abiotic stresses (Brotman et al., 2012; Hermosa et al., 2012; Rubio et al., 2017; Monte et al., 2019).

The effectiveness of plant pathogen suppression as well as growth promotion mediated by *Trichoderma* are species and strain dependent (Harman et al., 2004; Verma et al., 2007; Atanasova et al., 2013; Haddad et al., 2017; Sumida et al., 2018). Owing to the lack of studies exploring the biocontrol and biostimulant abilities of *Trichoderma* strains in association with cotton plants, this study aimed to investigate the potential of two novel indigenous Brazilian strains, *Trichoderma asperelloides* CMAA 1584 and *Trichoderma lentiforme* CMAA 1585, against *S. sclerotiorum* along with their role as cotton growth promoters.

## Material and methods

### Microorganisms

*Trichoderma asperelloides* CMAA 1584 (BRM 065723, GenBank accession ON542481) and *Trichoderma lentiforme* CMAA 1585 (BRM 065775, GenBank accession ON542480), both isolated from soil in Jaguariúna, SP, Brazil (22°43’43” S and 47°01’04” W), and deposited in the Collection of Microorganisms of Agricultural and Environmental Importance (CMAA) from Embrapa Environment (Jaguariúna, SP, Brazil), were used in these studies. These strains were reactivated and grown on potato-dextrose-agar medium (PDA; Acumedia Manufacturers^®^, Michigan, USA) in Petri dishes (9 × 1.5 cm) for 14 days at 25 ± 2 °C and 12:12 hours photoperiod. For preservation, 7-day-old sporulated colonies grown on PDA were cut into 5 mm pieces, placed in cryovials containing 1.5 mL of sterile solution of 20% (v/v) glycerol (Dinâmica^®^, São Paulo, SP, Brazil) prepared with double deionized water, and stored at –80 °C as stock cultures. Five-day-old PDA-grown cultures of these two *Trichoderma* strains were morphologically characterized based on colony growth aspects, conidiophores, and conidia size. Conidia size measurements were recorded with a light phase-contrast microscope (Olympus CS43 microscope and Olympus EP50 camera). The strains were identified phylogenetically using the translation elongation factor 1-α gene through direct comparison with data from reference type strains.

The plant pathogen *Sclerotinia sclerotiorum* CMAA 1105 (GenBank accession OM348513) was cultured on PDA in Petri dishes through myceliogenic germination from surface-sterilized sclerotia, and the newly-formed sclerotia were stored at 4 °C. This *S. sclerotiorum* strain was isolated in Jaguariúna, SP, Brazil (22°43’43” S and 47°01’04” W) in 1992, and was then deposited in the Collection of Microorganisms of Agricultural and Environmental Importance (CMAA) from Embrapa Environment (Jaguariúna, SP, Brazil). All fungal strains used in this study are registered under the Brazilian genetic heritage – SisGen – protocol A135E26.

### Ability of *Trichoderma* strains to solubilize phosphate

The ability of *Trichoderma* strains to solubilize inorganic phosphate (P) was evaluated by quantifying the solubilized P in liquid NBRIP (National Botanical Research Institute’s Phosphate) medium, which contained per liter: 10.0 g glucose, 5.0 g MgCl_2_.6H_2_O, 0.25 g MgSO_4_.7H_2_O, 0.2 g KCl and 0.1 g (NH_4_)_2_SO_4_ (Nautiyal, 1999). In the medium, 50 mL of K_2_HPO_4_ (10%) and 100 mL of CaCl_2_ (10%) were added to form an insoluble calcium phosphate (CaHPO_4_) precipitate. For inoculum production, 7-day-old sporulated cultures of each *Trichoderma* strain were rinsed with 10 mL of a sterile solution containing 0.04% polyoxyethylene sorbitan mono-oleate (Tween^®^ 80, Synth, SP, Brazil) and calibrated using a hemocytometer (improved Neubauer chamber, 400× magnification) under a microscope (DM 500, Leica Microsystems GmbH^®^, Germany) to provide a final inoculum size of 5 × 10^6^ conidia mL^-1^ in the medium. These liquid cultures were then incubated at 28 ± 1°C in an orbital rotary shaker (TE-1401, Tecnal^®^, Piracicaba, SP, Brazil) at 180 rpm for 5 days with a 12:12 hours photoperiod. The amount of calcium phosphate in the medium before inoculation of *Trichoderma* strains were approximately 150 µg mL^-1^. Aliquots of 1 mL were taken at the 5^th^ day and centrifuged at 7,000 rpm and 22 °C for 5 minutes to determine the concentration of soluble phosphorus, according to the colorimetric method described by Murphy and Riley (1962). The concentration of solubilized P in the supernatant was calibrated based on a standard curve of CaHPO_4_ (Sigma-Aldrich^®^, St. Louis, MO, USA) at concentrations of 0.5, 1.0, 2.0, 2.5, and 5.0 mg mL^-1^. The experiments were carried out with four biological repetitions to each fungal strain. Untreated control group (blank) was performed without the presence of microorganisms, whose values obtained were subtracted from those obtained in the presence of the fungal inoculum as a means to normalize the absorbance reads.

### Antifungal activity of *Trichoderma* strains against *S. sclerotiorum*

The ability of *Trichoderma* strains to antagonize *S. sclerotiorum* was evaluated by dual culture tests. Mycelial plugs (5 mm diameter) from the colony margin of an actively growing *Trichoderma* culture in PDA were placed on the edge of the Petri dish, and another plug of 7-day-old colony of *S. sclerotiorum* cultured on PDA was placed on the opposite side, maintaining 7 cm apart from colony discs. The plates were incubated at 25 ± 2 °C and the mycelial growth of both fungi was measured daily until *Trichoderma* strains have overgrown or surrounded the *S. sclerotiorum* colony. After 14 days of incubation under dual culturing, the antagonistic potential of *Trichoderma* strains inhibiting the pathogen’s growth was measured and the development of sclerotia was also evaluated. Furthermore, a diagrammatic scale proposed by Bell et al. (1982) was used to score the antagonistic capacity, where: 1 - *Trichoderma* overcomes the pathogen and grows in 100% of the plate; 2 - *Trichoderma* grows on at least 75% of the plate; 3 - *Trichoderma* and the pathogen colonize approximately 50% of the plate; 4 - The pathogen colonizes at least 75% of the plate and resists to *Trichoderma*; 5 - The pathogen completely overlaps *Trichoderma* and occupies the entire surface of the plate. As a control, Petri dishes inoculated only with the pathogen served as the reference to calculate the percent inhibition of pathogen’s colony growth exerted by *Trichoderma* strains. The experiment was performed with five biological replicates for each strain.

To assess the effect of volatile organic compounds (VOCs) released by *Trichoderma* strains on *S. sclerotiorum* mycelial growth, two Petri dish bottoms, one containing the pathogen and the other with a *Trichoderma* strain, all plated in the center and grown on PDA, were superimposed (Muthukumar et al., 2011). As a control, Petri dishes containing the pathogen were overlaid with another containing only PDA. These paired cultures were maintained in a growth chamber under the same environmental conditions described above. After 2 days of incubation, due to the rapid mycelial growth of *S. sclerotiorum*, the percentage of inhibition of the pathogen was assessed and further calculated by the equation: Inhibition (%) = (D_1_ – D_2_)/D_1_ × 100, where D_1_ represents the radial diameter of the pathogen in the control treatment, and D_2_ the radial diameter of the pathogen confronted with *Trichoderma*. The experiment was performed with five biological replicates for each strain.

### Parasitism of sclerotia of *S. sclerotiorum* by *Trichoderma* strains

The ability of both *Trichoderma* strains in parasitizing *S. sclerotiorum* sclerotia was evaluated in polypropylene boxes (11 cm × 11 cm × 3.5 cm) (Gerbox^®^) containing 200 g of a dystroferric dark red latosol, collected at Embrapa Environment and autoclaved at 121 °C for 60 minutes on three consecutive days. Dark-pigmented mature *S. sclerotiorum* sclerotia were produced in 500 mL Erlenmeyer flasks containing carrot and cornmeal, according to Garcia et al. (2012). Each autoclaved flask received three 5-mm-PDA discs of *S. sclerotiorum* mycelium, taken from the edge of a 7-day-old colony and incubated at 25 ± 2 °C. After 30 days of growth on carrot-cornmeal substrate, mature sclerotia were removed, placed on absorbent paper inside a laminar flow chamber, left drying for 24 hours, and then kept in a refrigerator at 4°C prior to using in bioassays. In each polypropylene box, 12 sclerotia were randomly distributed on the soil surface, and 10 mL suspensions containing 1 × 10^6^, 1 × 10^7^, and 1 × 10^8^ conidia mL^-1^ of each *Trichoderma* strain were evenly applied with a pipette over the soil surface. All groups were incubated for 15 days at 25 ± 2 °C with a photoperiod of 12:12 hours (Geraldine et al., 2013). A control group was set up with sterile distilled water. After 15 days of incubation, all sclerotia were removed from the soil, surface-sterilized with ethanol (70%) and sodium hypochlorite (2%) for 2 minutes, and subsequently rinsed three times in sterile distilled water prior to plating them on a selective media. Soft and disintegrated sclerotia due to colonization by *Trichoderma* strains were counted after slight pressure with a tweezer (Henis et al., 1983). Sclerotia viability was evaluated by incubating them on Neon medium (Napoleão et al., 2006) for 7 days at 25 ± 2 °C, then observing for the formation of a yellow halo around the sclerotia, which were deemed to be viable. The experiment was performed in a completely randomized design for each strain, with three treatments (inoculum size) and four biological replicates, in addition to a mock control treated only with water.

### Germination and vigor of cotton seeds treated with *Trichoderma*

Seeds of cotton cv. FM 975 WS^®^ provided by Instituto Mato-Grossense do Algodão (IMA, Mato Grosso, Brazil) were used in the interaction studies involving *Trichoderma* strains and cotton plants. The seeds were surface disinfected in 70% ethanol followed by 2% sodium hypochlorite solution for 2 minutes and washed in sterile distilled water three times. Surface-sterilized cotton seeds were soaked in an aqueous *Trichoderma* suspension containing 1 × 10^6^, 1 × 10^7^, and 1 × 10^8^ conidia mL^-1^ for 60 minutes, and then layered on a Petri dish to air-dry for 1 hour inside a laminar flow hood. *Trichoderma-*treated cotton seeds were sown in germitest^®^ paper (Cienlab Equipamentos Científicos Ltda, Campinas, SP, Brazil) using a roller system moistened with distilled water and incubated at 25 ± 2 °C. The experiment was set up in a completely randomized design with three treatments (inoculum concentrations) and four independent biological replicates, with 20 seeds each (i.e., total of 80 seeds per treatment). The number of germinated seeds was determined on the 4^th^ day after sowing, being expressed as a percentage of germinated seeds. Afterwards, the seedlings were dried in an oven at 105 °C until constant weight, and the vigor index was determined according to Abdul-Baki and Anderson (1973) by the equation: Vigor Index = Germination (%) × Seedling dry weight (g).

### Effect of *Trichoderma* on cotton growth

Cotton seeds cv. FM 975 WS^®^ were treated with crescent concentrations of *Trichoderma* conidia as described above and sown in rhizotron made of polyvinyl chloride (PVC) half-longitudinal tubes (100 cm height × 17.5 cm diameter), containing a mixture of a dystroferric dark red latosol and sand in a ratio of 2:1 (v/v). The soil exhibited the following chemical and physical attributes analyzed at 0 - 20 cm depth: pH in H_2_O = 4.3; OM = 32.3 g kg^-1^; P = 9.36 mg dm^-3^; Ca = 3.09 cmolc dm^-3^; Mg = 1.48 cmolc dm^-3^; K = 128.55 mg dm^-3^; SB = 4.95 cmolc dm^-3^; H + Al = 6.10 cmolc dm^-3^; t = 4.99 cmolc dm^-3^; V% = 44.54. In addition to seed treatment, 10 mL of the same conidial suspensions were applied in the planting furrow *via* drench at 15, 30 and 45 days after sowing (DAS). The experiment was set up in a randomized block design with three treatments (inoculum concentrations) and five independent biological replicates, in addition to mock cotton seeds as a control. The assay was carried out in a greenhouse for 60 days and the following growth parameters of the cotton plants were evaluated: height (14, 24, 31 and 55 DAS), root length (at 7, 14, 24 and 60 DAS) and leaf area of the first non-cotyledonary leaf (at 24 DAS), as described by Grimes and Carter (1969). At 60 DAS the following parameters were determined: stem diameter (2 cm above the soil surface), fresh and dry weights for both the aboveground portion and roots of the plants.

### Statistical analysis

Homogeneity of variances and normality tests were performed by Bartlett’s and Shapiro-Wilk tests. Data were fitted to linear models and analyzed by analysis of variance (ANOVA) using original data sets to identify significant differences between means of the treatments (Tukey’s test, *P* < 0.05). Statistical analyses were performed using Minitab^®^ software version 19.1.

## Results

### Morphological characterization of indigenous *Trichoderma* spp. strains

**Figure 1:**
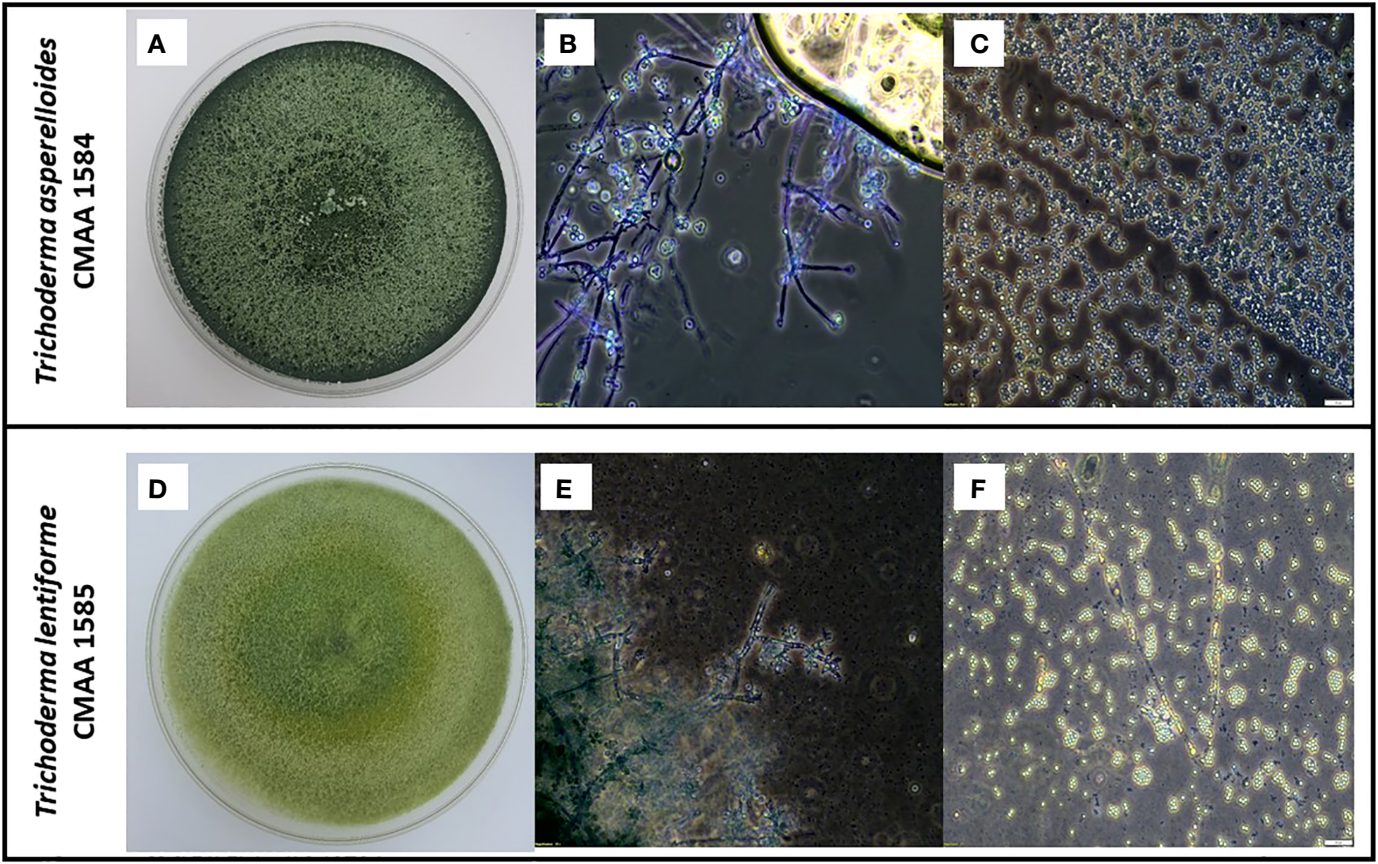
Morphological characterization of indigenous *Trichoderma asperelloides*, and *Trichoderma lentiforme* strains. **(A)** Five-day-old PDA-grown cultures of *T. asperelloides* (CMAA 1584); **(B)** Microscopic images showing conidiophores of CMAA 1584 (magnification at 200×); **(C)** Typical conidia of CMAA 1584 (magnification at 200×, scale bar = 20 µm); **(D)** Five-day-old PDA-grown cultures of *T. lentiforme* (CMAA 1585); **(E)** Microscopic images showing conidiophores of CMAA 1585 (magnification at 200×); **(F)** Typical conidia of CMAA 1585 (magnification at 200×, scale bar = 20 µm).

According to the phylogenetic analysis based on tef-alpha 1 gene, the strain CMAA 1584 was confirmed to be *Trichoderma asperelloides*, while the strain CMAA 1585 was identified as *Trichoderma lentiforme*. Purified monosporic cultures of these two *Trichoderma* spp. strains were very divergent from each other in terms of growth, color, conidial size, and conidiophores. As depicted in **Figure 1**, cultures of *T. asperelloides* CMAA 1584 exhibited profuse growth on PDA with dark green color when fully sporulated and forming ovoid conidia averaging 3.60 × 3.59 µm (length and width) with a resultant area estimated in 10.10 µm^2^ (standard error: ± 0.17 µm^2^, n = 20). When looking at *T. lentiforme* CMAA 1585 cultures, its sporulated colony assumed pale greenish color and produced conidia averaging 2.54 × 2.44 µm (length and width) with an estimated average area of 4.88 µm^2^ (standard error: ± 0.18 µm^2^, n = 20). The area size of *T. asperelloides* was noted to be twice larger than conidia of *T. lentiforme*. The morphological phenotypes and conidia sizes are consistent with values previously reported for these species (Samuels et al., 2010; Chaverri et al., 2015).

### Phosphate solubilization

*Trichoderma lentiforme* CMAA 1585 and *T. asperelloides* CMAA 1584 were both capable of solubilizing inorganic phosphate, resulting in about 31.7% and 5.2% of CaHPO_4_ remaining in the medium, respectively, in comparison to control (**Table 1**). Phosphate solubilization was significantly (*P* < 0.05) higher in NBRIP medium, in which *T. lentiforme* solubilized significantly more phosphate than *T. asperelloides* (**Table 1**).

**Table 1:** Phosphate solubilization by *Trichoderma asperelloides* CMAA 1584 and *Trichoderma lentiforme* CMAA 1585.

Strain	Phosphate solubilization (%)
CMAA 1584	5.2 ± 0.79 b
CMAA 1585	31.7 ± 4.08 a

Values represent means (± standard error) and when followed by the same letter do not differ significantly from each other (Tukey *p* < 0.05).

### Antifungal activity of *Trichoderma* strains against *S. sclerotiorum*

In general, volatile organic compounds (VOCs) released by *Trichoderma* strains significantly reduced (*P* < 0.05) the mycelial growth of *S. sclerotiorum* (**Table 2**). Compared to the control, VOCs emitted by *T. lentiforme* CMAA 1585 and *T. asperelloides* CMAA 1584 significantly reduced the growth rate of *S. sclerotiorum* by 55% and 53%, respectively (**Table 2**), albeit there was no difference between these *Trichoderma* strains in their ability to inhibit this pathogen by means of released VOCs.

**Table 2:** Antagonistic activity of *Trichoderma asperelloides* CMAA 1584 and *Trichoderma lentiforme* CMAA 1585 against Sclerotinia sclerotiorum by dual culture test and production of volatile organic compounds (VOCs).

Treatments	Volatile organic compounds		Dual culture		
	Growth rate(mm day^-1^)	Inhibition (%)	Growth rate(mm day^-1^)	Inhibition (%)	Number of sclerotia
CMAA 1584	23.6 ± 1.2 a (53%)	41.9 ± 2.7 a	27.4 ± 0.1 a (10%)	9.5 ± 0.4 a	0.6 ± 0.6 a
CMAA 1585	22.8 ± 1.5 a (55%)	43.8 ± 3.3 a	26.5 ± 0.6 a (13%)	12.2 ± 1.8 a	7.4 ± 1.5 b
Control	42.5 ± 0.0 b	–	30.5 ± 0.5 b	–	15.6 ± 1.6 c

Values in each column represent means (± standard error) and when followed by the same letter do not differ significantly from each other (Tukey *p* < 0.05).

Values between parentheses indicate the inhibition growth rate when compared to control.

**Figure 2:**
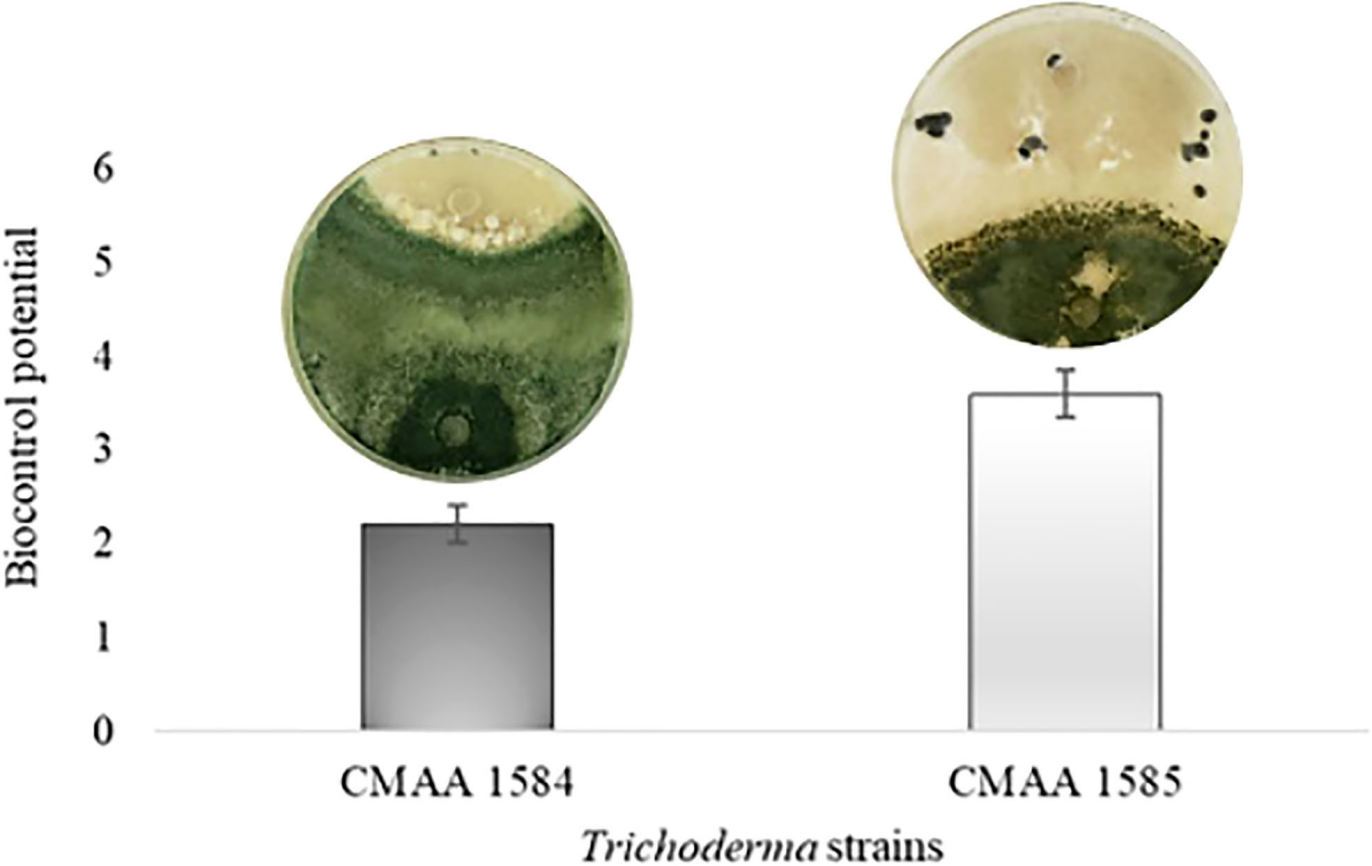
Biocontrol potential of *Trichoderma asperelloides* CMAA 1584, and *Trichoderma lentiforme* CMAA 1585 strains against Sclerotinia sclerotiorum according to Bell scale (Bell et al., 1982). Bars indicate means (± standard error).

In dual culture assay for direct confrontation, *T. asperelloides* CMAA 1584 and *T. lentiforme* CMAA 1585 reduced the growth rate (mm day^-1^) of *S. sclerotiorum* in 10% and 13%, respectively, when compared to control (**Table 2**) (*P* < 0.05). The inhibition of mycelial growth was 9.5% and 12.2% for *T. asperelloides* CMAA 1584 and *T. lentiforme* CMAA 1585, respectively (**Table 2**). Notably, *T. asperelloides* CMAA 1584 and *T. lentiforme* CMAA 1585 remarkably decreased by 96% and 47% the number of sclerotia formed by *S. sclerotiorum* colony in comparison to control, respectively (**Table 2**, *P* < 0.05). *Trichoderma lentiforme* CMAA 1585 received higher scores (3.6) when compared to *T. asperelloides* CMAA 1584 (2.2) according to Bell’s diagrammatic scale, indicating that the former was less aggressive in parasitizing sclerotia than the latter (**Figure 2**).

### Parasitism of sclerotia of *S. sclerotiorum* by *Trichoderma* strains

**Figure 3:**
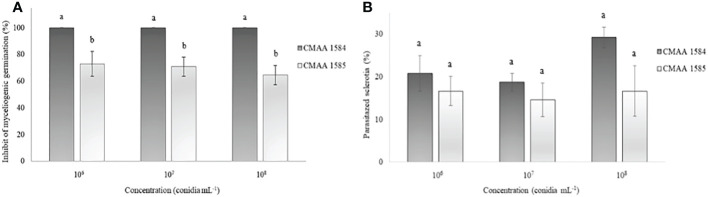
Ability of *Trichoderma asperelloides* CMAA 1584 and *Trichoderma lentiforme* CMAA 1585 strains to inhibit myceliogenic germination **(A)** and to parasitize **(B)** Sclerotinia sclerotiorum sclerotia by direct antagonism at different inoculum concentrations. *Bars indicate mean (± standard error). Means followed by the same letters do not differ from each other (Tukey test, *p* < 0.05).

The myceliogenic germination of sclerotia was significantly (*P* < 0.05) reduced by both *Trichoderma* strains (**Figure 3**). All concentrations of *T. asperelloides* CMAA 1584 colonized 100% of sclerotia and thus strongly inhibited the myceliogenic germination of all sclerotia (**Figure 3A**). *Trichoderma lentiforme* CMAA 1585, despite colonizing 100% of sclerotia, only 69% sclerotia were found ungerminated or non-viable based on the Neon selective medium test. The degradation of sclerotia did not reveal significant differences (*P* > 0.05) between *Trichoderma* strains for all concentrations tested (**Figure 3B**).

### Germination and vigor of cotton seeds treated with *Trichoderma*

*Trichoderma asperelloides* CMAA 1584 and *T. lentiforme* CMAA 1585 applied in cotton seeds, at 1 × 10^6^, 1 × 10^7^, and 1 × 10^8^ conidia mL^-1^, did not show any significant differences (*P* > 0.05) for seed germination and initial vigor of cotton seedlings, when compared to control (**Table 3**).

**Table 3:** Effect of *Trichoderma asperelloides* CMAA 1584 and *Trichoderma lentiforme* CMAA 1585 in cotton seeds germination and vigor index.

Treatments (conidia mL^-1^)	*T. asperelloides* CMAA 1584		*T. lentiforme* CMAA 1585	
	Germination (%)	Vigor index^1^	Germination (%)	Vigor index^1^
Control	92.5 ± 1.4 a	163.4 ± 1.5 a	92.5 ± 1.4 a	163.4 ± 1.5 a
1 × 10^6^	91.2 ± 2.4 a	165.6 ± 4.6 a	92.5 ± 1.4 a	164.2 ± 3.6 a
1 × 10^7^	88.7 ± 2.4 a	155.5 ± 8.3 a	92.5 ± 2.5 a	162.0 ± 3.8 a
1 × 10^8^	93.7 ± 3.1 a	163.6 ± 6.1 a	91.2 ± 1.2 a	164.7 ± 2.3 a

Values in each column represent means (± standard error) and when followed by the same letter do not differ significantly from each other (Tukey *p* < 0.05).

^1^Calculated according to Abdul-Baki and Anderson (1973) by the equation: Vigor Index = Germination (%) × Seedling Dry Weight (g).

### Effect of *Trichoderma* strains on cotton growth promotion

**Figure 4:**
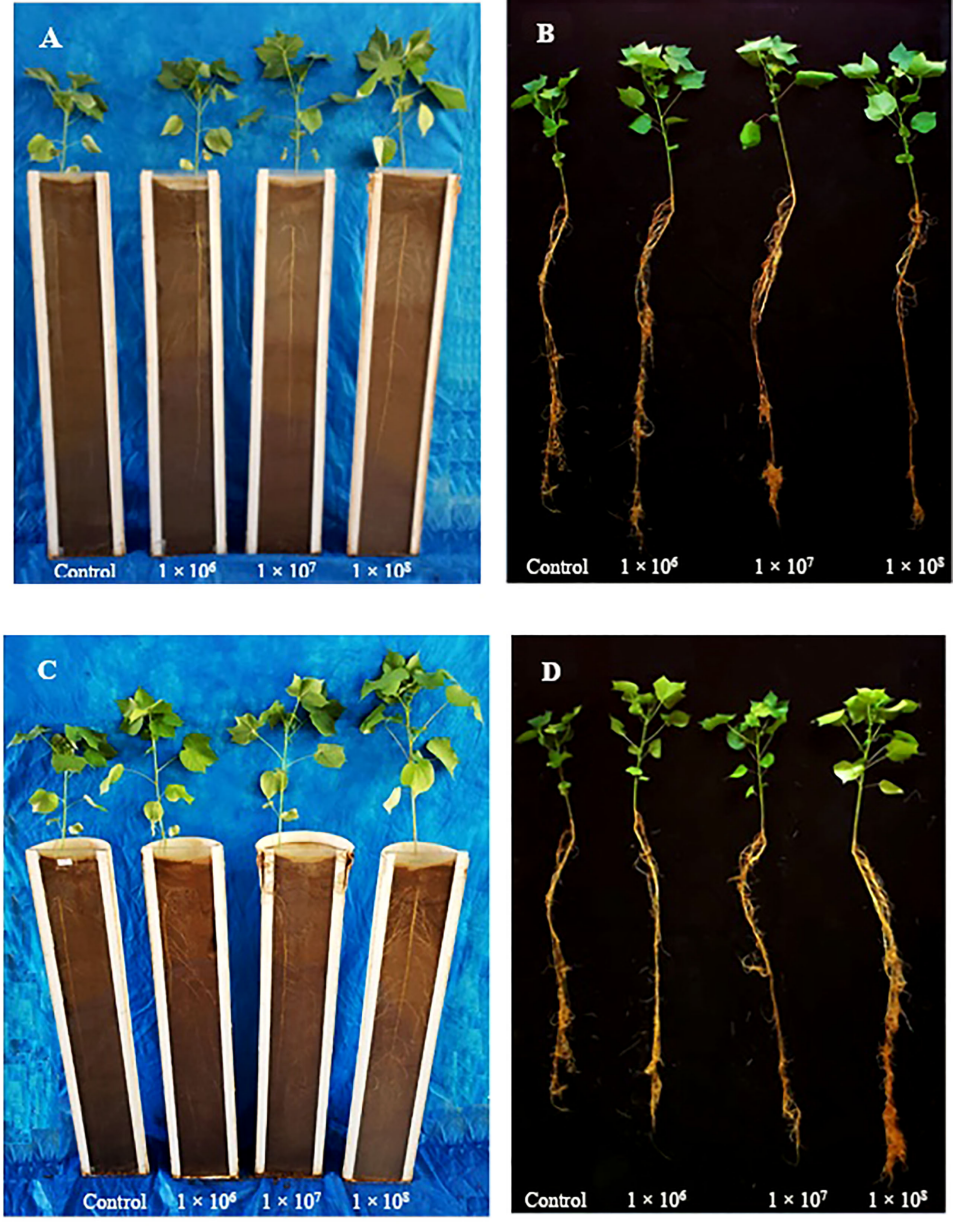
Cotton plants treated with *Trichoderma asperelloides* CMAA 1584 **(A, B)** and *Trichoderma lentiforme* CMAA 1585 **(C, D)** after 60 days of sowing in rhizotron.

**Figure 5:**
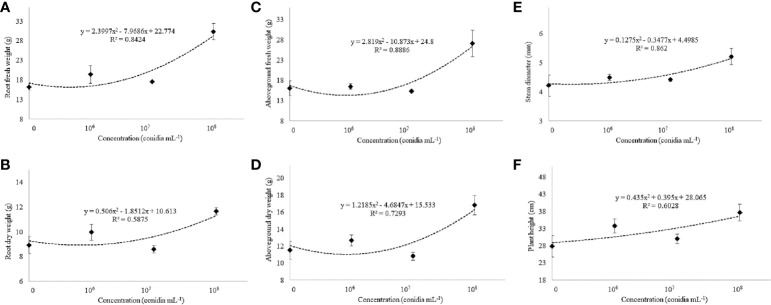
Fresh and dry weight of the root **(A, B)** and aboveground **(C, D)**, stem diameter **(E)**, and plant height **(F)** of cotton cultivar FM 975 WS^®^ with *Trichoderma lentiforme* CMAA 1585. Black diamond symbol represents means (± standard error) and the dashed line represents the fitted quadratic curve.

Under greenhouse conditions, growth of cotton plants derived from seeds coated with spores of *T. asperelloides* CMAA 1584 and *T. lentiforme* CMAA 1585 strains were compared with mock control plants. Notably, *T. lentiforme* CMAA 1585 outperformed *T. asperelloides* CMAA 1584 in promoting growth of cotton plants (**Tables 4**, **5**; **Figures 4**, **5**). Looking at *T. asperelloides* CMAA 1584, this strain incited significant cotton growth promotion only for leaf area (*P* < 0.05), inducing 98.9% and 42.0% larger leaf area than the mock control plants, when the fungus was applied to seeds at 1 × 10^7^ and 1 × 10^8^ conidia mL^-1^, respectively (**Table 4**, **Figures 5A, B**). Plant height, stem diameter, aboveground and root fresh and dry weights increased (*P* < 0.05) with the application of *T. lentiforme* CMAA 1585 at 1 × 10^8^ conidia mL^-1^ (**Table 5**, **Figure 4**). Notably, cotton plants derived from seeds treated with *T. lentiforme* CMAA 1585 at 1 × 10^8^ conidia mL^-1^ increased stem diameter, height, aboveground and root fresh and dry weights of 23.7%, 35.2%, 69.3%, 86.7%, 46.0%, and 30.4% (**Table 5**; **Figures 4**, **5**), when compared to control plants (*P* < 0.05), respectively.

**Table 4:** Leaf area, stem diameter, root length, plant height and fresh and dry weight of the root and aboveground of cotton plants treated with different concentrations of *Trichoderma asperelloides* CMAA 1584.

Treatment	Leaf area (cm^2^)	Stem diameter (mm)	Root fresh weight (g)	Root dry weight (g)	Aboveground fresh weight (g)	Aboveground dry weight (g)	Root length (cm)	Plant height (cm)
							60 DAS	55 DAS
Control	22.6 ± 3.7 a	4.2 ± 0.4 a	16.2 ± 0.9 a	8.9 ± 0.7 a	16.0 ± 1.8 a	11.5 ± 1.0 a	107.4 ± 2.2 a	27.8 ± 3.1 a
1 × 10^6^	25.7 ± 2.6 a	4.5 ± 0.2 a	16.9 ± 1.0 a	9.2 ± 0.4 a	15.4 ± 1.8 a	11.6 ± 0.6 a	108.4 ± 4.7 a	32.8 ± 1.5 a
1 × 10^7^	44.9 ± 2.0 b	5.0 ± 0.3 a	19.8 ± 1.0 a	10.4 ± 0.7 a	19.4 ± 1.9 a	13.1 ± 1.2 a	105.8 ± 0.8 a	33.3 ± 2.9 a
1 × 10^8^	32.0 ± 5.0 ab	5.0 ± 0.2 a	19.2 ± 1.4 a	9.5 ± 0.2 a	19.8 ± 2.0 a	12.9 ± 0.6 a	105.2 ± 2.5 a	34.2 ± 1.3 a
CV (%)^1^	24.6	14.0	14.0	13.1	26.2	17.5	6.4	16.7

Values in each column represent means (± standard error) and when followed by the same letter do not differ significantly from each other (Tukey *p* < 0.05). DAS, days after sowing.

1 Coefficient of variation.

**Table 5:** Leaf area, stem diameter, root length, height and fresh and dry weight of the root and aboveground of cotton plants treated with different concentrations of *Trichoderma lentiforme* CMAA 1585.

Treatment	Leaf area (cm^2^)	Stem diameter (mm)	Root fresh weight (g)	Root dry weight (g)	Aboveground fresh weight (g)	Aboveground dry weight (g)	Root length (cm)	Plant height (cm)
							60 DAS	55 DAS
Control	22.6 ± 3.7 a	4.2 ± 0.4 a	16.2 ± 0.9 a	8.9 ± 0.7 a	16.0 ± 1.8 a	11.5 ± 1.0 a	107.4 ± 2.2 a	27.8 ± 3.1 a
1 × 10^6^	26.1 ± 1.8 a	4.5 ± 0.1 ab	19.4 ± 2.3 a	9.9 ± 0.6 a	16.5 ± 0.7 a	12.7 ± 0.6 a	108.2 ± 3.8 a	33.8 ± 2.1 ab
1 × 10^7^	26.4 ± 2.8 a	4.4 ± 0.1 ab	17.5 ± 0.2 a	8.6 ± 0.3 a	15.4 ± 0.4 a	10.8 ± 0.4 a	112.0 ± 2.4 a	30.0 ± 1.2 ab
1 × 10^8^	33.7 ± 2.9 a	5.2 ± 0.3 b	30.3 ± 2.1 b	11.6 ± 0.3 b	27.1 ± 3.3 b	16.8 ± 1.1 b	110.2 ± 3.9 a	37.7 ± 2.4 b
CV (%)^1^	24.9	11.2	18.7	8.5	22.2	14.0	7.3	14.6

Values in each column represent means (± standard error) and when followed by the same letter do not differ significantly from each other (Tukey *p* < 0.05). DAS, days after sowing.

1 Coefficient of variation.

## Discussion

The present study reveals the ability of two indigenous Brazilian strains, *T. lentiforme* CMAA 1585 and *T. asperelloides* CMAA 1584 to solubilize inorganic phosphorus, a macronutrient of low availability in tropical soils. Furthermore, these strains are capable of emanating VOCs which inhibit the mycelial growth of *S. sclerotiorum*. In addition, these strains reduce the growth rate and total number of sclerotia of *S. sclerotiorum* in dual culture assay, and they inhibit the myceliogenic germination due to degradation of sclerotia through direct parasitism.

The difference in biocontrol performance between the *T. lentiforme* CMAA 1585 and *T. asperelloides* CMAA 1584 lies in their ability to suppress the myceliogenic germination of sclerotia, as reported in the present study (**Figure 2**), which may be related to secondary metabolites, including antifungal compounds, and the direct capacity of parasitism using an arsenal of well-known cuticle-degrading enzymes, where both mechanisms have been correlated with the virulence strategies employed by *Trichoderma* species (Harman et al., 2004; Geraldine et al., 2013; Monte et al., 2019). Previous studies have shown that *T. harzianum*, *T. koningii*, *T. pseudokoningii*, *T. koningiopsis*, *T. asperellum*, *T. atroviride*, and *T. virens* displayed excellent inhibitory effect on the myceliogenic germination of *S. sclerotiorum* in the range of 62% to 100%, when applied directly to the sclerotia (Haddad et al., 2017; Sumida et al., 2018). As noted in our study, evidence of interspecific variation in biocontrol efficacy among *Trichoderma* spp. is common and should be a key criterion to be incorporated into screening studies for biocontrol of plant pathogens.

There is a tremendous diversity among *Trichoderma* species and strains in their ability to produce and release biogenic volatile organic compounds (BVOCs) with remarkable roles in mediating plant growth and antagonism towards plant pathogens (Siddiquee et al., 2012; Contreras-Cornejo et al., 2014; Li et al., 2018). In this study, we noted that both of our *Trichoderma* strains imposed similar detrimental effects on *S. sclerotiorum* growth under *in vitro* conditions through emission of VOCs, whose compounds remain elusive. Given the importance of some VOCs emitted by *Trichoderma* playing pivotal roles in plant growth and biocontrol activity against plant pathogens, further research is needed to elucidate the emission profiles of VOCs by these *T. asperelloides* and *T. lentiforme* strains in view of providing new insights and applications of their metabolites in cotton growth enhancement and protection against white mold disease.

*Trichoderma asperelloides* CMAA 1584 showed a great potential for use in biological control of *S. sclerotiorum* as it inhibited 100% myceliogenic germination of all sclerotia exposed to their conidia, as well as decreasing the sclerotia formation by 96.1%, when compared to the mock control. According to Atanasova et al. (2013), *Trichoderma* spp. craft distinct strategies to combat and outcompete other host fungi. These authors observed host sensing in *T. atroviride* and *T. virens* through expression of genes involved in the attack, whereas *T. reesei* was keener to outcompete the pathogen for nutrients. Thus, screening studies for potential biocontrol candidates of *Trichoderma* can reveal interesting phenotypical traits between species and strains and differential pattern of gene expression linked to biocontrol during the parasitism process of targeted hosts (Atanasova et al., 2013; Troian et al., 2014). Our results strengthen the need to select the antagonist strain according to the desired targeted pathogen taking into account its biology and epidemiology in the crop system (Köhl et al., 2011; Bettiol et al., 2021).

Sclerotia that failed myceliogenic germination and were colonized by *Trichoderma* were classified as unviable, as this was the same criterion employed by Abdullah et al. (2008) and Görgen et al. (2009). Such mycotrophic lifestyle is one of the most remarkable antagonistic mechanisms expressed by *Trichoderma* spp. and is implicated in the direct attack of one fungal species to another (Sood et al., 2020). In this sequential process, the first step involves recognition by chemical cues of the targeted pathogenic fungus by *Trichoderma*, which then its hyphae attach and coil around the prey fungal hyphae (Harman et al., 2004), followed by the onset production of lytic enzymes that cause the dissolution of fungal cell walls (Druzhinina et al., 2011). Hence, it may be expected that antagonists with increased secretion of extracellular enzymes should be responsible for a more pronounced decline in the *S. sclerotiorum* inoculum levels in soil (Woo et al., 2006).

Among 20 strains of *Trichoderma* spp. evaluated for management of *S. sclerotiorum* in common beans, *T. asperellum* (cryptic sister species of *T. asperelloides*) exhibited the highest secretion of cell wall-degrading enzymes (CWDE) activity (Lopes et al., 2012). These data are consistent with those observed by Qualhato et al. (2013), where *T. asperellum* was effective against *Fusarium solani*, *Rhizoctonia solani* and *S. sclerotiorum*, and its antagonistic activity was associated with high activity of chitinase, β-1,3-glucanase and acid phosphatases. In our study, *T. asperelloides* CMAA 1584 displayed a great ability to inhibit myceliogenic germination and further degrade sclerotia of *S. sclerotiorum* by direct parasitism, outperforming *T. lentiforme* CMAA 1585 in this particular attribute. According to Geraldine et al. (2013), NAGase (N-β-acetylglucosaminidase) and β-1,3-glucanase enzymes play a key role in reducing the number of apothecia and the chain of events in the field that account for white mold severity, underlining the importance of these CWDEs in the control of white mold.

On the other hand, *T. lentiforme* CMAA 1585 demonstrates to be more suitable as a biostimulant due to its ability to boost growth of cotton plants (**Table 5**, **Figures 4** and **5**). The high phosphate solubilization in the soil (**Table 1**) displayed by this strain and better development of cotton roots are possible mechanisms associated with plant growth enhancement. Many authors have detailed the ability of *Trichoderma* spp. to modulate physiological, biochemical, and molecular mechanisms in a wide assortment of plants under various growth conditions (Hermosa et al., 2013; Rubio et al., 2014; Rubio et al., 2017; Elkelish et al., 2020), by the production phytohormones and a plethora of secondary metabolites (Jaroszuk-Ściseł et al., 2019).

Using *in vitro* bioassay, Contreras-Cornejo et al. (2009) showed that *T. virens* Gv29.8 and *T. atroviride* IMI206040 can synthesize indole-acetic acid [IAA] (and some of its derivatives), and suggests that the higher lateral root development observed in *Arabidopsis* wildtype plants is mediated by auxins. IAA synthesized by plant root-associated microorganisms can interfere with plant development by disturbing the auxin balance in plants, which can modify root architecture, increase root mass, and consequently, increase nutrient uptake by well-developed root system (Contreras-Cornejo et al., 2009). Sofo et al. (2011) reported that cherry rootstocks treated with *T. harzianum* commercial strain T-22 resulted in increased root and shoot growth by 76% and 61%, respectively. Furthermore, in mass spectrometry analyses these authors found that IAA and gibberellic acid (GA) levels were significantly increased by 40% and 143% in the roots, and by 49% and 71% in leaves, respectively.

Gravel et al. (2007) suggested that growth promotion, in tomato seedling, is associated with the reduced ethylene (ET) production resulting from a decrease in its precursor 1-aminocyclopropane-1-carboxylic acid (ACC), and/or through the ACC deaminase (ACCD) activity present in the microorganism. Another possible mechanism arises from increased plant tolerance to abiotic stresses and/or by mitigation of damages caused by the accumulation of reactive oxygen species (ROS) in stressed plants (Mastouri et al., 2010). Thus, it can be hypothesized that the absence of significant results (*P* > 0.05) in seed germination and seedling vigor index in our assay under laboratory conditions, using the germitest paper method, the growth promotion may be related to the absence of environmental stresses. When evaluating the germination and vigor of wheat seedlings (*Triticum aestivum* L.) after seed treatment with different strains of *Trichoderma* spp., Anjum et al. (2020) obtained results that corroborate this scenario, because in a greenhouse trial, the positive outcomes were significantly more expressive than those observed in *in vitro* test conducted in the laboratory. In the present study, although the germination index was similar for these *Trichoderma* strains in both growth conditions, cotton plants from the mock control group exhibited less growth in the greenhouse trial when compared with plants derived from the *Trichoderma* treatments, most likely due to the exposition of mock plants to suboptimal environmental conditions in contrast to higher resilience and improved growth promotion afforded by *Trichoderma* as a biological inoculant.

Both *Trichoderma* strains may be further tested under field conditions, but with different purposes. As such, we propose that *T. asperelloides* CMAA 1584 should be designated to control sclerotia of *S. sclerotiorum*, while *T. lentiforme* CMAA 1585 would assume a role as a biostimuant due to its ability to promote better growth of cotton plants. Overall, these selected *Trichoderma* strains are suitable for application in consortium targeting both pathogen control and growth promotion in cotton crops with a consequent contribution to diminishing the reliance on chemical fertilizers and fungicides.

## Data availability statement

The datasets presented in this study can be found in online repositories. The names of the repository/repositories and accession number(s) can be found in the article/supplementary material.

## Author contributions

WB, GM, and LG conceived and designed the laboratory and greenhouse experiments. LG, RC, and PS performed the laboratory and greenhouse experiments, and analyzed the data. WB and GM contributed with reagents/materials/analysis tools. CD provided critical analysis and editorial enhancements. All authors wrote the manuscript. All authors read and approved the final manuscript.

## Funding

This study was supported by Empresa Brasileira de Pesquisa Agropecuária (Embrapa SEG 20.19.02.006.00.00) and Coordenação de Aperfeiçoamento de Pessoal de Nível Superior - Brasil (CAPES) - Finance Code 001. Wagner Bettiol (CNPq 307855/2019-8) acknowledges Conselho Nacional de Desenvolvimento Científico e Tecnológico – CNPq for the productivity fellowship. This work was supported in part by the U.S. Department of Agriculture, Agricultural Research Service (Project Number: 5010-22410-024-00-D).

## Conflict of interest

The authors declare that the research was conducted in the absence of any commercial or financial relationships that could be construed as a potential conflict of interest.

## Publisher’s note

All claims expressed in this article are solely those of the authors and do not necessarily represent those of their affiliated organizations, or those of the publisher, the editors and the reviewers. Any product that may be evaluated in this article, or claim that may be made by its manufacturer, is not guaranteed or endorsed by the publisher.

## Author disclaimer

Any opinions, findings, conclusions, or recommendations expressed in this publication are those of the author(s) and do not necessarily reflect the view of the U.S. Department of Agriculture. The mention of firm names or trade products does not imply they are endorsed or recommended by the USDA over other firms or similar products not mentioned. USDA is an equal opportunity provider and employer.
